# Multitasking and the evolution of optimal clutch size in fluctuating environments

**DOI:** 10.1002/ece3.4364

**Published:** 2018-08-07

**Authors:** Ming Liu, Dustin R. Rubenstein, Siew‐Ann Cheong, Sheng‐Feng Shen

**Affiliations:** ^1^ Biodiversity Research Center Academia Sinica Taipei Taiwan; ^2^ Department of Entomology National Taiwan University Taipei Taiwan; ^3^ Department of Ecology, Evolution and Environmental Biology Columbia University New York New York; ^4^ Center for Integrative Animal Behavior Columbia University New York New York; ^5^ Division of Physics and Applied Physics School of Physical and Mathematical Sciences Nanyang Technological University Singapore Singapore; ^6^ Complexity Institute Nanyang Technological University Singapore Singapore

**Keywords:** bet‐hedging strategy, breeding season length, clutch size, environmental fluctuation, life‐history

## Abstract

Adaptive studies of avian clutch size variation across environmental gradients have resulted in what has become known as the fecundity gradient paradox, the observation that clutch size typically decreases with increasing breeding season length along latitudinal gradients, but increases with increasing breeding season length along elevational gradients. These puzzling findings challenge the common belief that organisms should reduce their clutch size in favor of additional nesting attempts as the length of the breeding season increases, an approach typically described as a bet‐hedging strategy. Here, we propose an alternative hypothesis—the multitasking hypothesis—and show that laying smaller clutches represents a multitasking strategy of switching between breeding and recovery from breeding. Both our individual‐based and analytical models demonstrate that a small clutch size strategy is favored during shorter breeding seasons because less time and energy are wasted under the severe time constraints associated with breeding multiply within a season. Our model also shows that a within‐generation bet‐hedging strategy is not favored by natural selection, even under a high risk of predation and in long breeding seasons. Thus, saving time—wasting less time as a result of an inability to complete a breeding cycle at the end of breeding season—is likely to be the primary benefit favoring the evolution of small avian clutch sizes during short breeding seasons. We also synthesize the seasonality hypothesis (pronounced seasonality leads to larger clutch size) and clutch size‐dependent predation hypothesis (larger clutch size causes higher predation risks) within our multitasking hypothesis to develop an integrative model to help resolve the paradox of contrasting patterns of clutch size along elevational and latitudinal gradients. Ultimately, our models provide a new perspective for understanding life‐history evolution under fluctuating environments.

## 1 INTRODUCTION

The adaptive nature of clutch size has received considerable attention in studies of life‐history evolution (Lima, 2009; Martin, 1987; Ricklefs, 1977). In general, longer breeding seasons are often suggested to favor smaller clutches because organisms can spread the risk of nest failure across multiple breeding attempts. Such a risk‐spreading strategy is often referred to as a conventional bet‐hedging strategy because it can lower the variance in breeding success within a season (Farnsworth, Simons, & Brawn, 2001; Griebeler, Caprano, & Böhning‐Gaese, 2010). Moreover, since this trade‐off between the mean and variance in fecundity over a short time period often occurs within an organism's lifetime, it constitutes a within‐generation bet‐hedging strategy (Hopper, Rosenheim, Prout, & Oppenheim, 2003; Sarhan & Kokko, 2007). Yet, theoretical studies have repeatedly pointed out that within‐generation bet‐hedging is only evolutionarily advantageous under restricted conditions, such as when populations are small or when they fluctuate in size (Hopper et al., 2003; Starrfelt & Kokko, 2012). Thus, it remains unclear whether laying multiple smaller clutches rather than fewer, larger ones is really a bet‐hedging strategy.

In addition to questions about risk spreading through laying multiple smaller clutches, within‐generation bet‐hedging also cannot explain the complex empirical patterns of avian clutch size variation that have been observed along latitudinal and elevational gradients (Pincheira‐Donoso & Hunt, 2017). Although clutch size is often found to increase with increasing latitude because breeding seasons are typically shorter in the temperate zone than in the tropics (Bennett & Owens, 2002; Jetz, Sekercioglu, & Böhning‐Gaese, 2008; Lack, 1954; Moreau, 1944), the opposite pattern is often found along elevational gradients (reviewed in Badyaev & Ghalambor, 2001; Boyce, Freeman, Mitchell, & Martin, 2015). In other words, clutch size *decreases* with increasing breeding season length across latitudinal gradients, but *increases* with increasing breeding season length along elevational gradients. These contrasting patterns—clutch size increasing with latitude but decreasing with elevation—have been referred to as the “fecundity gradient paradox” (Pincheira‐Donoso & Hunt, 2017).

Although breeding season length varies predictably with elevation and latitude, other major environmental sources of selection could vary in similar or opposite directions with increasing latitude and elevation. That is, nest predation risk is typically lower at higher latitudes and elevations (Boyle, 2008; McKinnon et al., 2010). According to the “clutch size‐dependent predation hypothesis,” if laying larger clutches attracts more predators (i.e., clutch size‐dependent predation), natural selection favors smaller clutch sizes in lower latitudes and elevations (Lima, 2009; Skutch, 1949). Similarly, if the risk of nest predation is independent of clutch size, then the conventional view of bet‐hedging—which predicts that a longer breeding season leads to smaller clutch sizes—cannot explain the fecundity gradient paradox because breeding seasons are shorter at both higher latitudes and elevations. In addition, seasonality of food availability is higher at higher latitudes because increased seasonality in resources causes higher winter mortality, which results in greater food availability per individual in the spring. According to the “seasonality hypothesis,” Ashmole (1963) predicted that clutch sizes will be larger at higher latitudes. However, it remains unclear whether the same pattern holds in higher elevations. In short, we still have a surprisingly limited understanding of how breeding season length, food availability, and environmental unpredictability shape the evolution of clutch size, as well as other life‐history traits (Hau, Wikelski, Gwinner, & Gwinner, 2004; Rubenstein, 2007).

Although many theoretical models have considered the impacts of breeding season length on clutch size evolution, most, if not all, of these studies use a fixed number of breeding attempts to represent the length of breeding season. However, the number of breeding attempts is not equivalent to breeding season length because (1) it may take longer to recover and begin subsequent nesting attempts after laying larger clutches (Deerenberg, de Kogel, & Overkamp, 1996; Drent & Daan, 1980) and, (2) nest failure can occur at different times within a breeding attempt, which influences both the duration of a nesting attempt and the recovery time for beginning the next breeding attempt. Thus, the number of breeding attempt should be dynamically adjusted according to the timing of nest failure and the clutch size. In other words, it should take longer to recover if nest failure occurs later in a breeding attempt or if individuals lay larger clutches, because of greater energy investment in the current breeding attempt. As a result, the possible time‐saving strategy under severe time constraints for multiple breeding attempts within a season has not been explicitly considered when modeling the evolution of clutch size.

We propose an alternative hypothesis, the “multitasking hypothesis,” as a time‐saving strategy to explain the evolution of clutch size under reproductive bouts of varying length and in fluctuating environments. Multitasking in humans is characterized by frequent switching between different tasks (reviewed in Kiesel et al., 2010). Frequent task‐switching often has high costs, such as reduced efficiency (Arrington & Logan, 2004; Rubinstein, Meyer, & Evans, 2001) or performance (Rogers & Monsell, 1995), when tasks are more difficult or complex. Despite these potential costs, time saving is a major benefit of multitasking because it enables individuals to complete more than one task in a timely manner. Animals face similar trade‐offs between focusing on a single task with high efficiency and simultaneously performing multiple activities with lower efficiency that take longer to complete than a single task. Despite being a topic of great interest in the human psychology literature (Kiesel et al., 2010; Monsell, 2003), the role of multitasking in shaping the evolution of life‐history strategies has largely been neglected.

An example of multitasking in animals is brood overlap, the simultaneous provisioning of multiple broods, which occurs in some fishes and birds (Burley, 1980). Since by definition brood overlap creates short intervals between breeding attempts within a season, individuals typically save time through multitasking (Burley, 1980). Nonetheless, the energetic costs of producing successively overlapping broods can be substantial (Møller, 2007) because successive breeding requires that individuals frequently switch tasks between recovery (e.g., foraging to gain essential nutrients storage) and investing in reproduction, including provisioning previous young, building new nests, and laying eggs (Ridley & Raihani, 2008). As an analogy, income breeders that acquire energy and breed simultaneously can also be viewed as using a multitasking strategy when compared to capital breeders that focus on storing energy before each breeding bout (Houston, Stephens, Boyd, Harding, & McNamara, 2007). Importantly, focusing on a single task and multitasking should be considered as two ends of a continuum marked by variation in the time spent on one task before switching to another.

Here, we employ the concept of multitasking to model the evolution of clutch size evolution in a fluctuating environment. Producing a small clutch is considered to be a multitasking strategy if it requires less energy storage prior to breeding, thereby enabling organisms to switch between storing energy and breeding more frequently. In other words, smaller clutch sizes allow organisms to renest more quickly and conserve time. However, producing small clutches can also be energetically inefficient since organisms cannot forage or pursue other activities as much while breeding. We model the evolution of multitasking by explicitly considering the impacts of breeding season length and the degree of environmental fluctuation on clutch size evolution using individual‐based and analytical modeling approaches. Furthermore, to help resolve the fecundity gradient paradox, we also test previously proposed hypotheses regarding clutch size evolution, including (1) the bet‐hedging hypothesis (Farnsworth et al., 2001; Griebeler et al., 2010), (2) Skutch's clutch size‐dependent nest predation hypothesis (Lima, 2009; Skutch, 1949), and (3) Ashmole's seasonality hypothesis (Ashmole, 1963; McNamara, Barta, Wikelski, & Houston, 2008). Ultimately, our model both synthesizes previous hypotheses and highlights the importance of a time‐saving strategy for understanding the evolution of clutch size.

## 2 MATERIALS AND METHODS

### 2.1 Model overview and the individual‐based model

**Figure 1:**
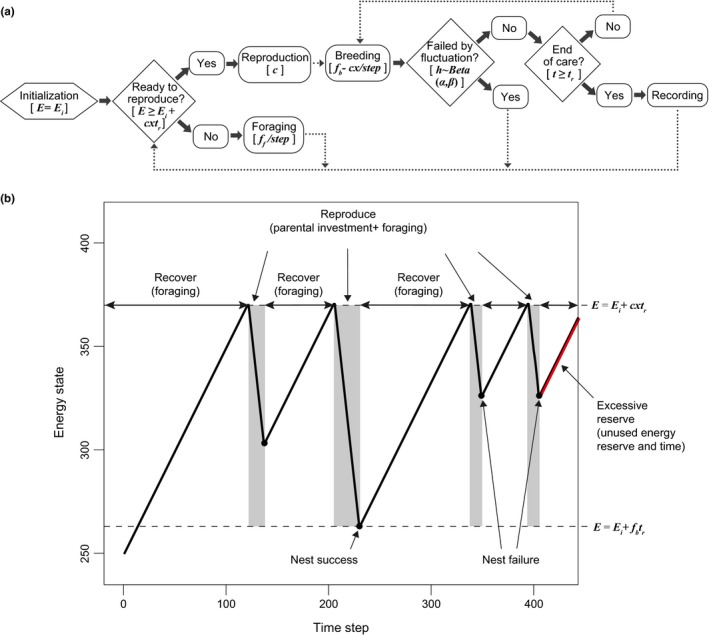
Process overview of model and diagram of energy dynamics. (a) In the flow chart, dotted arrows indicate proceeding to the next time step, and solid arrows represent a sequential event in the same step. We list the key variables or parameters of each event, which are expressed inside brackets. (b) The change of energy state with time is plotted in the diagram, where reproduction periods are shown in the gray area and recovering periods are shown in the white. Note that only successful nests last *t*_*r*_ steps and, thus, the energy reserve will decline to the lowest point. Finally, the excessive reserve is the time and energy wasted at the end of the breeding season due to the time constraint [Colour figure can be viewed at http://wileyonlinelibrary.com]

We consider only (1) foraging events to raise energy reserves and (2) breeding events to produce offspring that reduce energy reserves. We assume that an individual will be able to both invest energy (i.e., breeding) and acquire energy (i.e., foraging) while it is breeding, as well as gain energy (i.e., foraging) during the intervals between breeding attempts (reproduce and recover, respectively, see Figure 1). Within a breeding season, the decisions of “when to start breeding” is considered to be an energy‐based decision. In this simple model, energy reserves must exceed the cost of successful breeding before producing a clutch (i.e., *E* ≥ *E*_*i*_ + *cxt*_*r*_). Specifically, *E*_*i*_ is the basic energy reserve and *cxt*_*r*_ is the product of the number of offspring produced in each breeding attempt (i.e., the clutch size) (*c*), the cost per offspring (*x*), and the time required for breeding successfully (*t*_*r*_). Hence, the amount of energy and time required to commence breeding are positively correlated to clutch size (e.g., Eden, Horn, & Leonard, 1989; Møller, 2007; Nwaogu, Dietz, Tieleman, & Cresswell, 2017; Smith, Källander, & Nilsson, 1987). For simplicity, we also assume that clutch size is a fixed strategy and remains the same for each individual in each nesting attempt (see Supporting Information Figure S1 for reducing clutch size throughout the season). We denote the basic foraging efficiency per foraging event as *f*_*f*_. Since we assume that there is a trade‐off between breeding and foraging, the foraging efficiency in each breeding event (*f*_*b*_) declines as offspring number increases (i.e., $f_{b}=f_{f}*\left(1-c/c_{\max}\right)$, where the maximum clutch size of each attempt is *c*_max_).

The probability of nest failure due to predation, severe weather, or other similar factors at each time step is *h*, and is independent of clutch size. If nest failure occurs, all offspring within a clutch die. Specifically, we set the probability distribution of nest failure rate, *h*, as a beta distribution: $h∼Beta\left(\alpha ,\beta \right),\;\alpha =h_{m}/h_{v},\;\beta =\left(1-h_{m}\right)/h_{v}$, where *h*_*m*_ is the mean, and *h*_*v*_ is related to variance. A beta distribution is ideal for sampling probabilities because it offers values that are constrained between 0 and 1. We use a beta distribution that is convex and positively skewed (i.e., 1 < *α *< *β*). Although nest failure in each time step is often considered to be relatively rare, even with a relatively low probability of nest failure in each time step, the overall breeding success rate can still be low. For example, if it takes 25 days to incubate and feed nestlings until fledging, a 90% daily survival rate will result in only a 7.2% likelihood of fledging young. Therefore, we only use beta distributions with a mean below 0.5 to describe the daily nest failure rate. In particular, we randomly select a value sampled from the distribution (*h*) to represent the current environmental conditions in each time step of a breeding event. This value is then compared to a random number between 0 and 1; if *h* is higher than the randomly drawn number, the attempt fails and the individual goes back to the foraging/breeding decision. Note that the breeding season length in our model is a relative concept such that more time steps indicate a longer breeding season and vice versa, but a step does not represent an actual day or year. Nevertheless, our model can be modified to fit specific life history of organisms by adjusting the rate of energy intake, the predation risk, and the length of the breeding period or season. Additionally, we also model a scenario of clutch size‐dependent predation by setting higher nest failure rates for larger clutch sizes.

When offspring survive to the end of breeding event (*t*_*r*_ time steps), that attempt is considered to be successful. In such cases, the number of fledglings (i.e., the clutch size of focal individual, *c*) is recorded before going back to the foraging/breeding decision. Because there is no exit to this loop process, an individual will keep trying to breed until the breeding season terminates (i.e., the *T* ^th^ time step). After the breeding season has ended, the recorded numbers of fledglings are summed to obtain the fitness of a given strategy. We simulate the fitness of a strategy *n* times and discuss the mean and variance in fecundity of each breeding strategy under a season of finite length, *T*. In summary, this model acquires efficiency of foraging energy, amount of investment in reproduction, and compares fledgling numbers of each breeding strategy (all variables and parameters are summarized in Table 1).

**Table 1:** Summary of model parameters

Name	Value	Description
*T*	500^b^	Length of breeding season
*E, E*_*i*_	250^a^, 250	Amount of energy reserve and its basic (lowest) value
*c, c*_max_	1^b^, 10	Clutch size (reproductive strategy), and its upper limit
*f*_*f*_ *, f*_*b*_	1*, f*_*f*_ *(1*‐c/c*_max_)	Foraging efficiency during a foraging and breeding event, respectively
*x*	1	Cost of caring for offspring at each time step
*t*_*r*_	24	Duration of successful breeding event
*h, h*_*m*_ *, h*_*v*_	0^a^, 0.03, 0.01^b^	Nest failure rate and shape parameters, $h∼Beta\left(\alpha ,\beta \right),\;\alpha =\frac{h_{m}}{h_{v}},\beta =\frac{\left(1-h_{m}\right)}{h_{v}}.$
*n*	10,000	Number of simulation run

a These variables are updated in each step. Only initial values are shown here.

b These values can vary depending on the goal of the simulation.

## 3 RESULTS

### 3.1 Model assumptions: Trade‐offs between multitasking and concentrating on a single task

**Figure 2:**
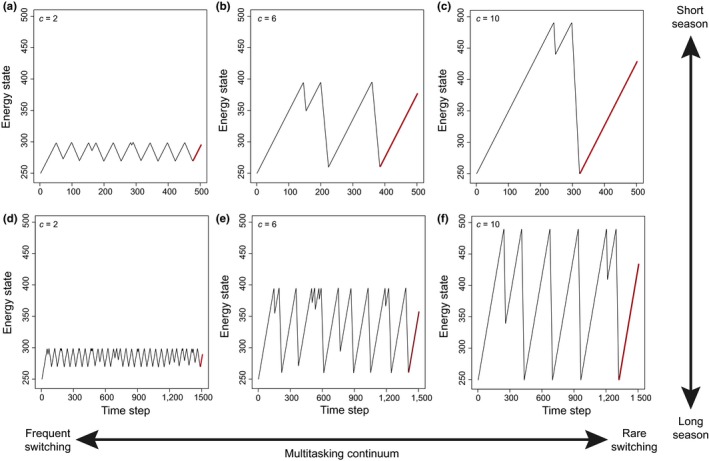
Time series of energy dynamics. Different clutch size strategies (i.e., *c *=* *2, 6, 10) represent the multitasking continuum from (a) to (c) and (d) to (f), respectively, as switching frequency is higher in under the small clutch strategy. (a–c) represent a short breeding season length with 500‐time steps and (d, e) a long breeding season length with 1,500‐time steps. Red lines indicate the excessive reserve (see text for details) [Colour figure can be viewed at http://wileyonlinelibrary.com]

In this section, we examine the behavior of the model and the energy dynamics of each clutch size strategy. Based on the assumption that producing a larger clutch requires a larger energy reserve (*cxt*_*r*_), the small clutch size strategy requires a shorter foraging time to accumulate enough energy reserve to initiate breeding. This means that the number of breeding attempts decreases with an increase in clutch size (Figure 2). Thus, producing smaller clutches is a strategy that is similar to the multitasking end of the continuum because task‐switching—switching between accumulating energy reserve by foraging and spending energy on breeding—occurs more frequently. We also assume that whether a nest will survive to the next time step depends on the environmental conditions of the current time step, something that is determined by the stochastic function (i.e., beta distribution) in this model. The remaining energy reserve therefore depends on the timing of breeding termination. If nest failure occurs soon after the start of breeding, the loss of energy reserve will be minor. However, if breeding is successful, energy reserves will decline to their lowest point. As a result, we do not assume a fixed number of breeding attempts, as most previous models have done (Farnsworth et al., 2001; Pöysä, Pesonen, Tim, & Jonathan, 2007). Instead, the number of breeding attempts in a season depends on the probability of nest failure.

**Figure 3:**
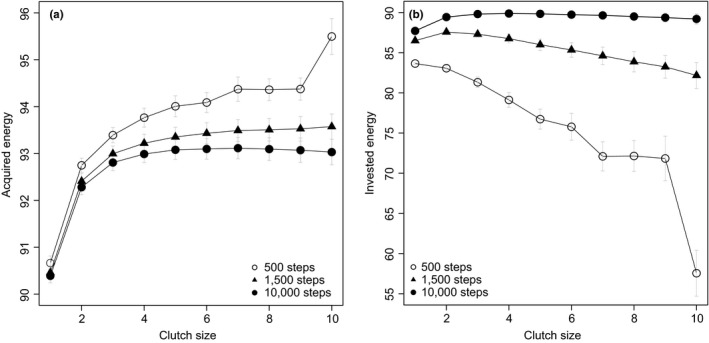
Standardized acquired energy and invested energy of various reproductive strategies (*c*) with respect to season length (*T*). Acquired energy (a) is the sum of energy gained from foraging (including *f*_*f*_ and *f*_*b*_) during the breeding season; invested energy (b) is the sum of energy spent in reproduction during the breeding season, regardless of its success or failure. Each line indicates a breeding season length. Means and standard deviations are standardized by season lengths (i.e., divided by *T* and $\sqrt{T}$, respectively) to avoid unfair comparison. Each data point represents the average value from 10,000 simulations

The cost of multitasking is further determined by the sum of energy income (i.e., energy reserves gained by foraging) that the multitasking strategy suffers from a greater loss in efficiency. Indeed, the small clutch size strategy has a lower total energy income compared to the large clutch size strategy (Figure 3a), despite the former foraging more during breeding (i.e., *f*_*b*_). This result can once again be explained by the frequency of task‐switching. That is, although organisms can retain certain foraging abilities during breeding, these abilities never reach the efficiency of concentrating on foraging alone (i.e., *f*_*f*_). Therefore, more frequent switching to breeding results in a reduced overall foraging efficiency.

### 3.2 Breeding season length—multitasking or bet‐hedging?

**Figure 4:**
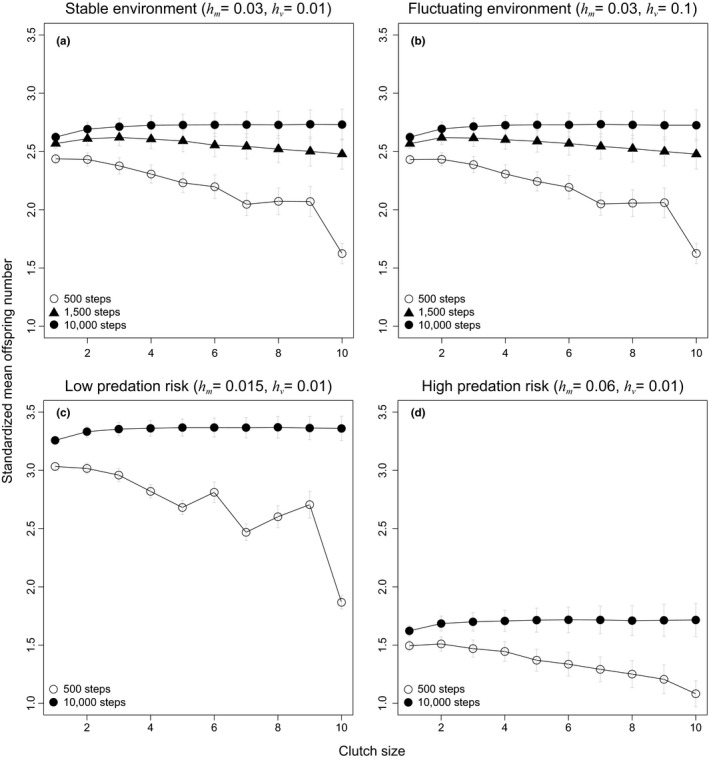
The effect of breeding season length clutch size evolution: testing the multitasking and bet‐hedging hypotheses. Since the two hypotheses predict opposite trends on the evolution of clutch size, we verify their predictions by manipulating the variance (a, b) and mean (a, c, d) of the nest failure rate (see Table 1 for more details). These changes in the environment resemble different types of environmental fluctuations. Specifically, each line or type of points are the average number of offspring produced of each breeding strategy (i.e., clutch size) in the same breeding season length (e.g., *T *=* *500, 1,500, 10,000 time steps). Mean offspring numbers are standardized by season lengths, and error bars indicate half the size of standard error. In addition, all data are standardized in the same way as Figure 3, and each point is collected from 10,000 repeated simulations

Does lower foraging efficiency imply lower offspring output? We further explore the impact of breeding season length on clutch size evolution. Interestingly, we find that the average fitness of the large clutch size strategy is *lower* than that of the small clutch size strategy during short breeding seasons, but slightly *higher* during long breeding seasons, which clearly contradicts the conventional view of the bet‐hedging hypothesis (Figure 4a). Here we assume that a larger clutch size does not directly lead to higher nest predation risk, but the results are qualitatively similar as long as the negative impacts of a larger clutch size are not exceedingly high (see later section for details). Similarly, the large clutch size strategy also expends less energy than the small clutch size strategy in short breeding seasons (Figure 3b, 500 steps), though more in long breeding seasons (Figure 3b, 10,000 steps). Although it initially seems counterintuitive for individuals to adopt the large clutch size strategy—forage more but breed less—in short breeding seasons, clues about the cause behind this result can be seen in the energy dynamics (Figure 2). That is, a longer preparation time and a lower number of breeding attempts under the large clutch size strategy make the strategy more likely to waste vast amounts of time and energy at the end of season (Figures 2 and 3). In other words, the larger energy demands associated with laying large clutches cause breeders to lose more time and energy by the end of season compared to those that lay small clutches, which never requires storing such a large amount of energy prior to breeding. This wasted time and energy, which we refer to as an “excessive reserve,” reduces the efficiency in energy use for the large clutch size strategy (Figures 2c, 3a), thus making it suboptimal during short breeding seasons. Conversely, as the length of the breeding season increases, the size of this wasted excessive reserve remains, but overall energy income escalates, resulting in a decline in the excessive reserve to income ratio. This means that the excessive reserve is trivial and the large clutch size strategy is again more efficient than the small clutch size strategy. Ultimately, this excessive reserve to income ratio may be an important mechanism underlying clutch size evolution in birds.

### 3.3 Nest failure rate: Mean and variance of environmental conditions

We then consider the influences of the mean and variance in nest failure rates on the evolution of clutch size. We find that different levels of variance have little effect on the evolution of clutch size (Figure 4a,b). Although this result might seem puzzling, it can be explained by the fact that at the level of genotype, having many individuals with the same genotype (or breeding strategy) reduces the variance of fitness of the genotype in the population. Similarly, the mean nest failure rate alone does not favor large or small clutch size (see also Figure 4c,d), regardless of the length of breeding season. Again, these results are not consistent with the conventional view of bet‐hedging hypothesis, though they are predicted by a within‐generation bet‐hedging hypothesis (Hopper et al., 2003; Starrfelt & Kokko, 2012).

### 3.4 Clutch size‐dependent predation hypothesis

**Figure 5:**
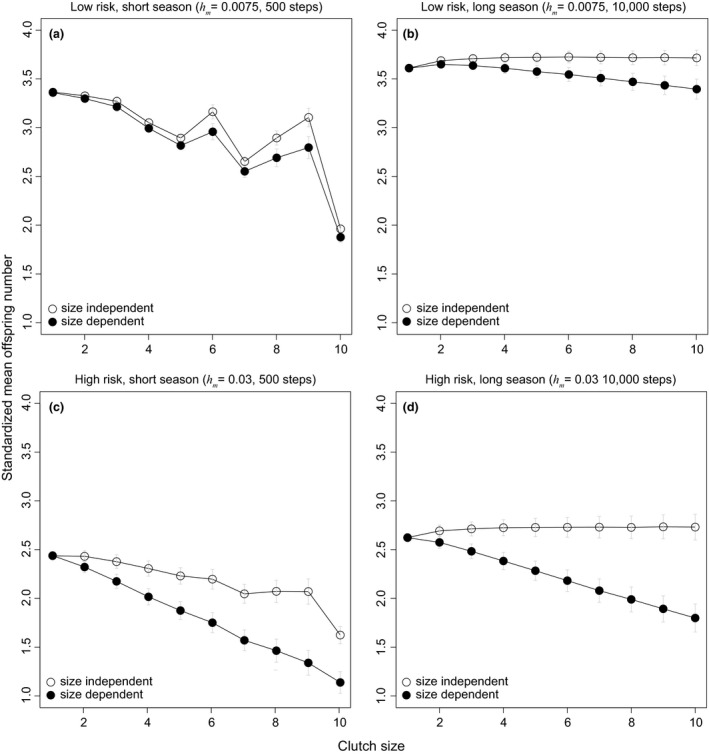
Clutch size‐independent and clutch size‐dependent predation. Number of offspring under (a) low predation risk and short breeding seasons, (b) low predation risk and long breeding seasons, (c) high predation risk and short breeding seasons, and (d) high predation risk and long breeding season. *T* and *h*_*m*_ represent the length of the breeding season and the nest failure rate, respectively. Other parameter values and method of standardizing data are the same as those described in Figure 4

We assume that clutch size does not influence nest failure rate in the models described above (i.e., clutch size independent predation). Here we incorporate into the model Skutch's (1949), clutch size‐dependent predation hypothesis, which suggests that predation rates might be higher when clutches are larger. We find that selection favors smaller clutches if larger clutches result in reduced nest success, even during long breeding seasons, a result that is opposite to predictions of the clutch size independent predation hypothesis (Figure 5d). Furthermore, the effect of clutch size‐dependent predation is more pronounced when the predation risk is high, as suggested by Skutch (comparing Figure 5c,d to Figure 5a,b).

### 3.5 Seasonality hypothesis

**Figure 6:**
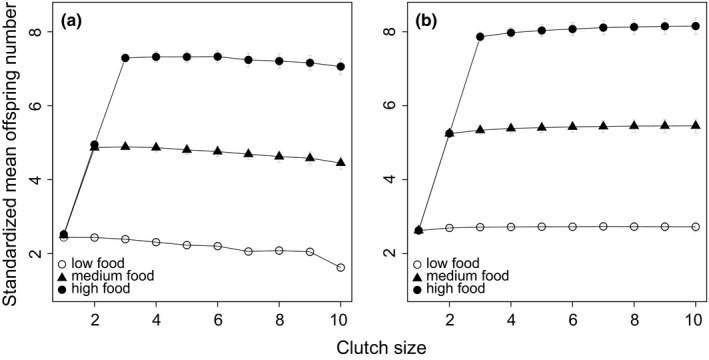
Food availability and clutch size. Ashmole's food availability hypothesis is tested in both short (a, 500 steps) and long (b, 10,000 steps) breeding seasons. Furthermore, three levels of food availability (*f*_*f *_= 1, 2, 3 for low, medium, and high, respectively) are set for the two season lengths. Note that since the mean of the nest failure rate is 0.03, lines with low food availability resemble the results in Figure 4a

Finally, we investigate Ashmole's seasonality hypothesis by altering food availability in the model (i.e., foraging efficiency, *f*_*f*_ and *f*_*b*_). Intuitively, we find that greater food availability favors larger clutch sizes even during shorter breeding seasons (Figure 6). This occurs because individuals can recover more quickly after a breeding attempt, since food availability is greater. However, as clutch size becomes larger, breeding season length, but not the food availability, becomes the key factor constraining the evolution of larger clutch size. As a result, offspring number decreases slightly under the larger clutch size strategy in short season, but clutch sizes increase slightly in long season.

### 3.6 The analytical approximation of the multitasking model

**Figure 7:**
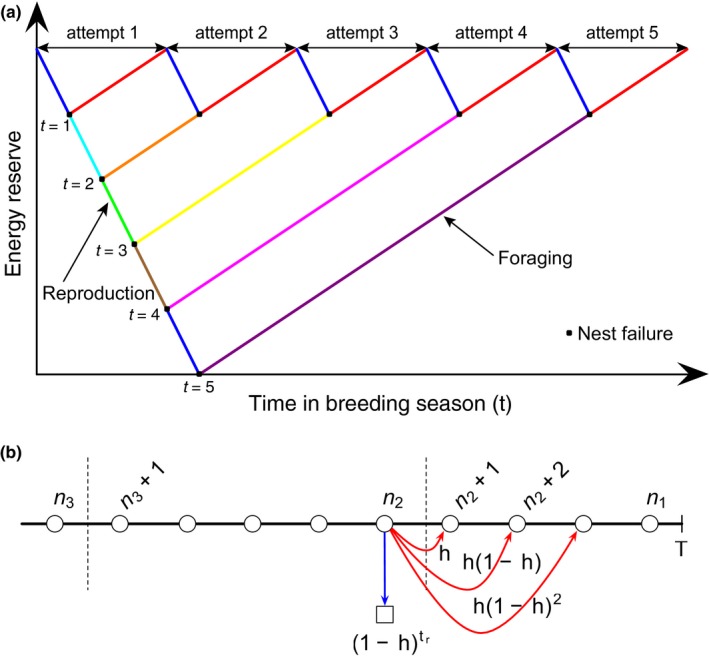
Discrete starting times of breeding attempts (a) and breeding attempts at the end of the breeding season (b). If a breeding attempt fails after *k* time steps, then taking into account the time needed to bring its energy reserve up to *E* = *E*_*i*_+*cxt*_*r*_/*f*_*f*_, an individual will start the next breeding attempt at the same time as another individual that has failed *k* times after a single time step. Breeding attempts can therefore only start at a discrete set of times *t*_*n*_ = *cxt*_*r*_/*f*_*f*_+*ncx*/*f*_*f*_, *n* = 0, 1, 2, …. In contrast, in figure (b), $t_{n_{1}}$ is the last time in the breeding season for a breeding attempt to start (but never complete), $t_{n_{1}}$ is the last time in the breeding season for a breeding attempt to start, and end successfully, and $t_{n_{s}}$ is the last time in the breeding season for a breeding attempt to start, and should it fail, be able to restart at or before $t_{n_{2}}$. The probabilities for a breeding attempt starting at $t_{n_{2}}$ to restart at $t_{n_{2}}+1$ (failing after one time step), $t_{n_{2}}+2$ (failing after two time steps), $t_{n_{2}}+3$ (failing after three time steps), …, and successfully completing the breeding after *t*_*r*_ time steps [Colour figure can be viewed at http://wileyonlinelibrary.com]

Given the simplicity of the simulation model of multitasking, we further derive analytical results for the model to develop deeper insights into this time‐saving strategy. To do this, let us simplify the model further, to make our calculations easier. Suppose that an individual gains *f*_*f*_ units of energy during each time step when it is not breeding, but instead of gaining $f_{b}=f_{f}\left(1-c/c_{\max}\right)$ units of energy every time step when it breeds, it is merely expending *x* units of energy every time step. Let us further suppose that starting at a threshold energy reserve of *E*_*i*_ at the beginning of the breeding season, the individual must first spend *cxt*_*r*_/*f*_*f*_ time steps accumulating energy reserve before starting its first breeding attempt. Thereafter, breeding attempts can only begin at discrete sets of time $t_{n}=cxt_{r}/f_{f}+n\left(1+cx/f_{f}\right)$ because, as illustrated in Figure 7a, the time it takes for an individual that failed after *k* time steps to start the next breeding attempt is the same as that for another individual that failed *k* times after one time step.

In this discrete set of starting times, there are three times of which we must take note. The first, $n_{1}=\left\lfloor \left(T-cxt_{r}/f_{f}\right)/\left(1+cx/f_{f}\right)\right\rfloor$, is associated with the last time $t_{n_{1}}$ in the breeding season that a breeding attempt can start. Here ⌊z⌋ is the greatest integer function, whose value is the integer part of the real number *z*. The second, $n_{2}=\left\lfloor \left(T-cxt_{r}/f_{f}=t_{r}\right)/\left(1+cx/f_{f}\right)\right\rfloor$, is associated with the last time $t_{n_{2}}$ in the breeding season that a breeding attempt can start *and* end successfully before the breeding season is over. The third, $n_{3}=\left\lfloor \left(T-cxt_{r}/f_{f}-t_{r}\right)/\left(1+cx/f_{f}\right)-t_{r}\right\rfloor$, is associated with the last time $t_{n_{s}}$ in the breeding season that a breeding attempt can start *and* if it fails, *be able to restart* at or before $t_{n_{2}}$. These three times are shown in Figure 7b. Also shown in Figure 7b are the probabilities that a breeding attempt starting at *t*_*n*_ will restart at *t*_*n*+1_ (failing after one time step), *t*_*n*+2_ (failing after two time steps), *t*_*n*+3_ (failing after three time steps), …, or ultimately succeeding after *t*_*r*_ time steps. The probability for a breeding attempt to fail after *k * ≤  *t*_*r*_ time steps is

$$p\left(k\right)={\left(1-h\right)}^{k-1}h,$$

because the probability for the nest to survive for the first (*k*−1) time steps is (1−*h*) for each time step and that for the nest to fail on the *k*th time step is *h*, assuming *h* is a time‐independent constant. Consequently, the probability for a nest to survive *t*_*r*_ time steps (and the breeding attempt successful) is

$$q={\left(1-h\right)}^{t_{r}}.$$

If the environmental nest failure rate *h* is time‐independent, these *transition probabilities* are the same for all starting times. This is true of the starting times $\left\{t_{n_{3}+1},\ldots ,t_{n_{2}}\right\}$, which are all starting times that can produce one final breeding success, but never again in the breeding season. If a breeding attempt starting at $t_{n_{3}+1}<t_{n}<t_{n_{2}}$ fails after a small number of time steps, it is possible for a new breeding attempt to start at or before $t_{n_{2}}$ and be successful. When this happens, however, we cannot consider *t*_*n*_ to be the last complete breeding attempt. In order for the breeding season *after* the breeding attempt starting *t*_*n*_ to be wasted, this breeding attempt must not fail early. In fact, for a breeding attempt starting at *t*_*n*_ to be probabilistically independent of breeding attempts starting at other times, this attempt must succeed after *t*_*r*_ time steps. When this happens, the energy recovery part of the breeding attempt (i.e., *T*−*t*_*n*_−*t*_*r*_) will be wasted. To compute the average time wasted in a breeding season, we observe that *T*−*t_n_* will be wasted for *n*_2_ < *n* ≤*n*_1_ with unit probability, while *T*−*t*_*n*_−*t*_*r*_ will be wasted for *n*_3_ + 1 ≤ *n *≤ *n*_2_ with probability ${\left(1-h\right)}^{t_{r}}$.

At the zeroth level (of season length), if we do not worry about starting times *t*_*n*_ in the range *n*_2_ < *n* ≤*n*_1_, the average time wasted would be

$$T-\left\langle t_{n}\right\rangle -t_{r}\approx \frac{t_{r}}{2}\left(1+\frac{\mathrm{cx}}{f_{f}}\right),$$

from the midpoint of $\left(t_{n_{3}+1},t_{n_{2}}\right)$, with a standard deviation of

$$\frac{t_{r}}{12}\left(1+\frac{\mathrm{cx}}{f_{f}}\right).$$

Therefore, at this zeroth level approximation, the time wasted and its standard deviation increases linearly with clutch size *c*. Compared to the duration *T* of the breeding season, this time wasted is a fraction

$$\frac{t_{r}}{2T}\left(1+\frac{\mathrm{cx}}{f_{f}}\right),$$

which means that the loss in breeding success of an individual with clutch size *c* would be

$$\rho_{\max}\frac{t_{r}}{2T}\left(1+\frac{\mathrm{cx}}{f_{f}}\right),$$

where *ρ*_max_ is the maximum breeding success possible. Here, we see that the loss in breeding success increases as *T* becomes shorter. At this level of approximation, we see also that the average time wasted and its standard deviation are independent of the environmental nest failure rate *h*, and so give the same result whether *h* is time‐independent or stochastic.

To improve on this approximation, we compute the average time wasted as

$$\frac{\left(T-t_{n_{1}}\right)\cdot 1+\cdots +\left(T-t_{n_{2}-1}\right)\cdot 1+\left(T-t_{n_{2}}-t_{r}\right)\cdot {\left(1-h\right)}^{t_{r}}+\cdots +\left(T-t_{n_{3}+1}-t_{r}\right)\cdot {\left(1-h\right)}^{t_{r}}}{\left(n_{2}-n_{1}\right)\cdot 1+\left(n_{3}-n_{2}\right)\cdot {\left(1-h\right)}^{t_{r}}},$$

which can be written as the weighted average

$$w\frac{t_{r}}{2}+\left(1-w\right)\frac{t_{r}}{2}\left(1+\frac{\mathrm{cx}}{f_{f}}\right),$$

where

$$w=\frac{\left(n_{2}-n_{1}\right)\cdot 1}{\left(n_{2}-n_{1}\right)\cdot 1+\left(n_{3}-n_{2}\right)\cdot {\left(1-h\right)}^{t_{r}}}.$$

Further, using the fact that

$$\frac{n_{3}-n_{2}}{n_{2}-n_{1}}=\frac{t_{r}\left(1+cx/f_{f}\right)}{t_{r}}=1+\frac{\mathrm{cx}}{f_{f}},$$

we see that the average time wasted in the breeding season is

$$\frac{1}{1+\left(1+\frac{\mathrm{cx}}{f_{f}}\right){\left(1-h\right)}^{t_{r}}}\frac{t_{r}}{2}+\frac{\left(1+\frac{\mathrm{cx}}{f_{f}}\right){\left(1-h\right)}^{t_{r}}}{1+\left(1+\frac{\mathrm{cx}}{f_{f}}\right){\left(1-h\right)}^{t_{r}}}\frac{t_{r}}{2}\left(1+\frac{\mathrm{cx}}{f_{f}}\right).$$

For this exact treatment when *h* is time‐independent, both the average time wasted and its standard deviation are functions of *c* and *h*, but we expect the general behavior to be similar to that for the zeroth‐order approximation. Because the first term in the average time wasted is not probabilistic, we expect the standard deviation of the average time wasted to simply be

$$\frac{t_{r}}{12}\frac{\left(1+\frac{\mathrm{cx}}{f_{f}}\right){\left(1-h\right)}^{t_{r}}}{1+\left(1+\frac{\mathrm{cx}}{f_{f}}\right){\left(1-h\right)}^{t_{r}}}\left(1+\frac{\mathrm{cx}}{f_{f}}\right).$$

**Figure 8:**
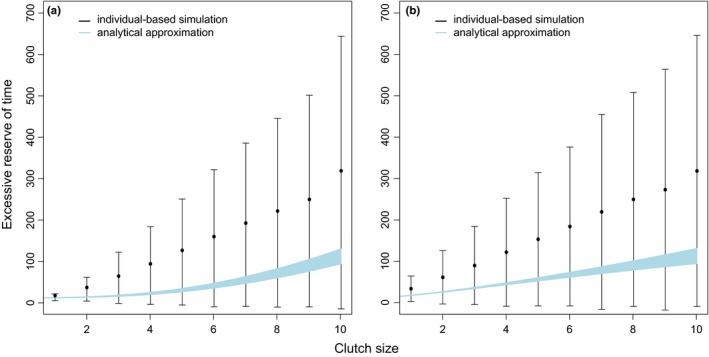
Comparison of individual‐based simulations and analytical approximations. The mean ± standard deviation of excessive reserve of time, where black segments are from the individual‐based model and the blue area is from analytical calculation. Excessive reserve of time is defined as the time wasted after the last reproduction attempt by the end of breeding season. Both with (a) and without (b) foraging during breeding are plotted, where *f_b_* is *f*_*f*_ *(1*‐c/c*_max_) and 0, respectively (see analytical approximation in text and Figure 7 for details). In simulation data, each clutch size is repeated 10,000 times under 10,000‐step season length setting [Colour figure can be viewed at http://wileyonlinelibrary.com]

Finally, when $h∼Beta\left(\alpha ,\beta \right)$ is stochastic, we note that

$$\langle {\left(1-h\right)}^{t_{r}}\rangle =\left\langle 1-\left(\begin{array}{c} t_{r}\\ 1 \end{array}\right)h+\left(\begin{array}{c} t_{r}\\ 2 \end{array}\right)h^{2}-\cdots \right\rangle =1-\left(\begin{array}{c} t_{r}\\ 1 \end{array}\right)\left\langle h\right\rangle +\left(\begin{array}{c} t_{r}\\ 2 \end{array}\right)\left\langle h^{2}\right\rangle -\cdots ,$$

which is larger than ${\left(1-\langle h\rangle \right)}^{t_{r}}$ for small *h* because the contribution from $\left\langle h^{2}\right\rangle =h_{m}^{2}+h_{v}^{2}$ dominates. Also, we can bring $f_{b}=f_{f}\left(1-c/c_{\max}\right)$ back into the discussion by replacing *x* by $x-f_{f}\left(1-c/c_{\max}\right)$. This introduces an additional clutch size dependence into the average time wasted, but does not change the overall dependence on *t*_*r*_/*T*. Finally, we compare the results of average wasted time, which is the excessive reserve of time (see Figures 1 and 2), in both individual‐based simulations and analytical zeroth‐order approximations. As shown in Figure 8, the results of the two models have similar trends where the excessive reserve increase with clutch size, demonstrating the generality of this key result.

## 4 DISCUSSION

Our models—both analytical and individual‐based simulation—demonstrate that large clutch sizes are favored in longer breeding seasons because higher foraging efficiencies enable individuals to accumulate a greater total energy income for breeding. In contrast, although small clutch sizes result in lower foraging efficiency due to frequent task‐switching between recovering and breeding, such a strategy is actually favored in shorter breeding seasons because less time and energy are wasted under severe time constraints for multiple breeding attempts within a season (i.e., there is less excessive reserve). Thus, our results demonstrate that saving time during constrained breeding seasons is the primary benefit favoring the evolution of small clutches sizes (a multitasking strategy). This finding generates opposite predictions to the conventional view of the bet‐hedging hypothesis (Farnsworth et al., 2001; Griebeler et al., 2010), as well as complementary predictions to Skutch's clutch size‐dependent nest predation hypothesis (Lima, 2009; Skutch, 1949), and Ashmole's seasonality hypothesis (Ashmole, 1963; McNamara et al., 2008) (see below for details).

To our knowledge, our multitasking hypothesis provides the first explanation for helping to resolve the fecundity gradient paradox (Pincheira‐Donoso & Hunt, 2017) (Table 2). According to our models, the pattern of smaller clutches at higher elevations can be explained by the “excessive reserve effect” that occurs in shorter breeding seasons. That is, since parental investment generally has a negative physiological effect on parental body condition and energy reserve (Blount, Houston, & Møller, 2000; Blount, Houston, Surai, & Møller, 2004; Møller, 1993, 1997; Schantz, Bensch, Grahn, Hasselquist, & Wittzell, 1999), longer renesting intervals are expected for larger clutch sizes due to higher parental energy and nutrient requirements. In other words, laying large clutches requires more time to complete a breeding bout than does laying small clutches. As a result, the excessive reserves of time and energy, such as not enough time for initiating and/or completing a new clutch, will occur more frequently when species lay large clutches, especially when breeding seasons are short. Since nest starvation at the end of the breeding season‐a mechanism that actually constrains the length of breeding season‐will also occur more frequently when clutches are large, the large clutch size strategy will be selected against when breeding seasons are shorter, if all other things are equal.

**Table 2:** Comparison of the four hypotheses. These four hypotheses are not mutually exclusive as they may have a combination of effects to the fitness of each breeding strategy (i.e., clutch size)

Name	Description	Prediction	Simulation results	Literature review
Multitasking hypothesis	Switching frequency among clutch sizes produce a trade‐off between the size of the excessive energy reserve and overall efficiency	Larger clutches in longer breeding seasons	Same as prediction (Figure 4a)	This paper
Conventional bet‐hedging hypothesis	Select against variance, and related to number of nest attempts, as well as the mean and variance of nest failure rate	a. Smaller clutches in longer breeding seasons	Opposite trend to prediction (Figure 4a)	Farnsworth et al. (2001); Griebeler et al. (2010)
		b. Higher failure rate or variance favors smaller clutch sizes	Almost no effect (Figure 4)	Doligez and Clobert (2003); Griebeler et al. (2010)
Within‐generation bet‐hedging hypothesis	The within‐generation variance of reproductive output has little effect on clutch size evolution	No relationship between mean nest failure rate or variance and clutch size	Same as prediction (Figure 4)	Hopper et al. (2003), Starrfelt and Kokko (2012)
Clutch size‐dependent predation hypothesis	Higher predation in larger clutches	Small clutches are advantageous, especially under high predation risk	Same as prediction (Figure 5)	Skutch (1949); Lima (2009)
Seasonality hypothesis	Food is the limiting factor of clutch size	Larger clutches when food is more abundant	Same as prediction (Figure 6)	Ashmole (1963), McNamara et al. (2008)
Synthetic model	Multiple mechanisms, including multitasking, clutch size‐dependent predation, and seasonality hypotheses, can work in concert to affect clutch size.	A longer breeding season at lower elevations selects for larger clutch sizes, which explains the elevational pattern of clutch size; whereas less pronounced seasonality and a higher degree of clutch size‐dependent predation at lower latitudes lead to smaller clutches	Same as prediction (Supporting Information Figure S2)	This paper

Laying small clutches is often considered to be a risk‐spreading strategy of bet‐hedging that is thought to be favored during longer breeding seasons (Farnsworth et al., 2001; Griebeler et al., 2010), and when the risk of nest predation is high (reviewed in Lima, 2009). However, some theoretical studies have argued that laying multiple, small clutches is essentially a within‐generation bet‐hedging strategy that only operates under fluctuating and/or small population sizes (Hopper et al., 2003; Starrfelt & Kokko, 2012). In agreement with the above theoretical arguments, our models demonstrate that mean and variance in nesting success (caused by predation and/or environmental fluctuation) have little impact on clutch size evolution, and thus, that small clutch sizes are unlikely to be explained by risk‐spreading and the conventional view of bet‐hedging. Our models even suggest that longer breeding seasons can favor the evolution of larger clutches if laying larger clutches is more energetically efficient. Thus, our results are consistent with previous empirical findings showing that if predation risk is considered to be independent of clutch size (i.e., larger clutches do not cause the substantial increase in predation rate), higher predation risk will not lead to smaller clutch sizes (Ricklefs, 1977).

How, then, can we explain the well‐established pattern that clutch size increases with increasing latitude (Bennett & Owens, 2002; Jetz et al., 2008)? We explored two influential but nonmutually exclusive hypotheses: Skutch's clutch size‐dependent predation hypothesis (Lima, 2009; Martin, 1996; Skutch, 1949) and Ashmole's seasonality hypothesis (Ashmole, 1963; Griebeler et al., 2010; McNamara et al., 2008; Ricklefs, 1980). In support of Skutch's hypothesis, we found that smaller clutches are favored under higher predation risk. This prediction holds even in longer breeding seasons where laying smaller clutches is more time‐consuming and less efficient. Much empirical evidence suggests that larger clutches attract more predators because of increased parental activity around the nest to incubate eggs or care for offspring (Martin, Scott, & Menge, 2000; Møller, 1990). Thus, the smaller clutches observed in tropical environments are likely due to the higher relative success rate under high predation risk. In contrast, as Ashmole suggested, temperate regions might have relatively more abundant food resources per individual for breeding due to higher mortality in the winter (seasonality hypothesis). Our model also found that higher food availability can select for larger clutches, even under short breeding seasons. This occurs because higher food availability lowers the preparation and recovery times necessary for the larger clutch size strategy, and, thus, the cost of laying a larger clutch is minimized. Thus, the fecundity gradient paradox could be resolved by simultaneously considering the relative importance of time‐saving, food availability, and clutch size‐dependent predation in the evolution of clutch size (see Supporting Information Figure S2 for a synthetic model incorporating these hypotheses).

In conclusion, although the concept of multitasking has been largely ignored in studies outside of humans, it may prove useful for examining life‐history evolution. Multitasking is generally found to be costly and inefficient in humans because frequent task‐switching incurs costs, such as interference costs when having previously performed a different task or expecting to perform a different task subsequently interferes with current performance (Allport, Styles, & Hsieh, 1994; Kiesel et al., 2010; Koch, Schuch, Vu, & Proctor, 2011; Steinhauser & Hübner, 2009), or as a task‐set reconfiguration cost where performing unfamiliar new tasks require additional time and/or energy to prepare (Meiran, 2000; Rubinstein et al., 2001). Although time savings is often assumed to be the primary benefit of multitasking, both empirical support and theoretical support for this idea are scarce, even in humans. Our models on clutch size evolution generate a novel mechanism demonstrating that the decreasing excessive reserve—the wasted time and energy at the end of breeding season—when time is constrained is a major benefit of multitasking (i.e., laying small clutches). The apparent obstacle that prevents us from fully resolving the fecundity gradient paradox with multitasking hypothesis is that we do not know the exact mechanisms causing the contrasting clutch size patterns along the elevational and latitudinal gradients. Nevertheless, we help identify potentially key factors—such as breeding season length, seasonality, and the relationship between clutch size and predation risk—and underlying mechanisms that may or may not work (e.g., bet‐hedging hypothesis) to facilitate the design of future empirical studies that can help to further explain existing results. We believe that the theory of multitasking can potentially be applied to wide range of biological phenomena beyond clutch size evolution, especially those cases that have previously been considered to be bet‐hedging strategies, such as multibrooding, clutch overlap, income breeding, and brood parasitism. Ultimately, our models provide a new perspective for understanding life‐history evolution and adaptation under fluctuating environments.

## CONFLICT OF INTEREST

No competing interests.

## AUTHOR CONTRIBUTION

S.‐F.S. conceived the idea. M.L.& S.‐F.S. constructed the individual‐based model. S.‐A.C. constructed the analytical approximation. All authors designed the simulations, analyzed the data, and wrote the article.

## DATA ACCESSIBILITY

All source codes in R and C and data are available at https://github.com/mingpapilio/Codes_Multitasking.
