# Supplementary figures and images for: Multitasking and the evolution of optimal clutch size in fluctuating environments
Source: Ecol Evol. 2018 Aug 7;8(17):8803–17. doi: 10.1002/ece3.4364 (PMC6157677; doi:10.1002/ece3.4364)

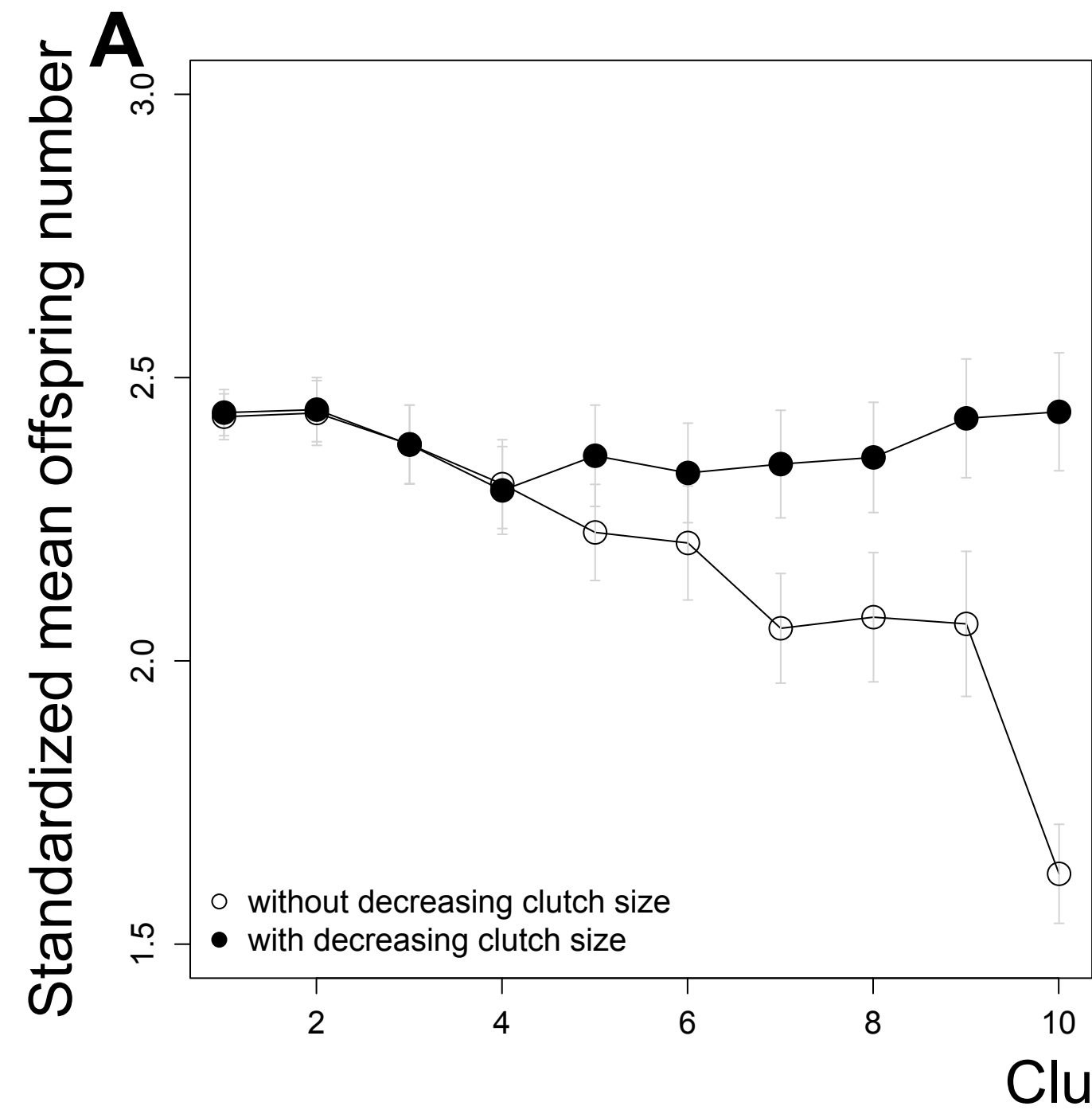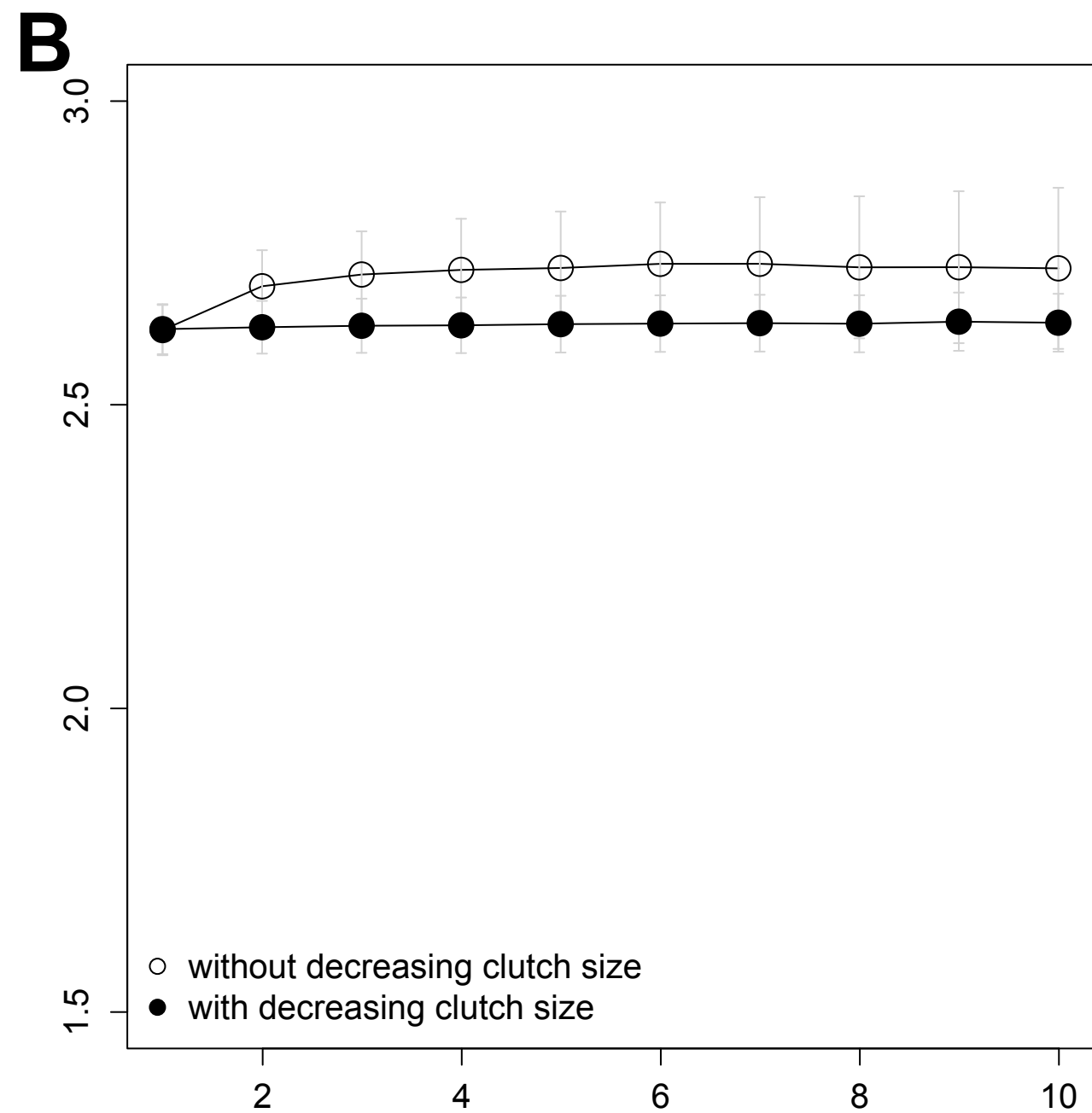

Supplement: Supplementary file 1 [file ECE3-8-8803-s001.pdf]

Standardized mean offspring number

**A**

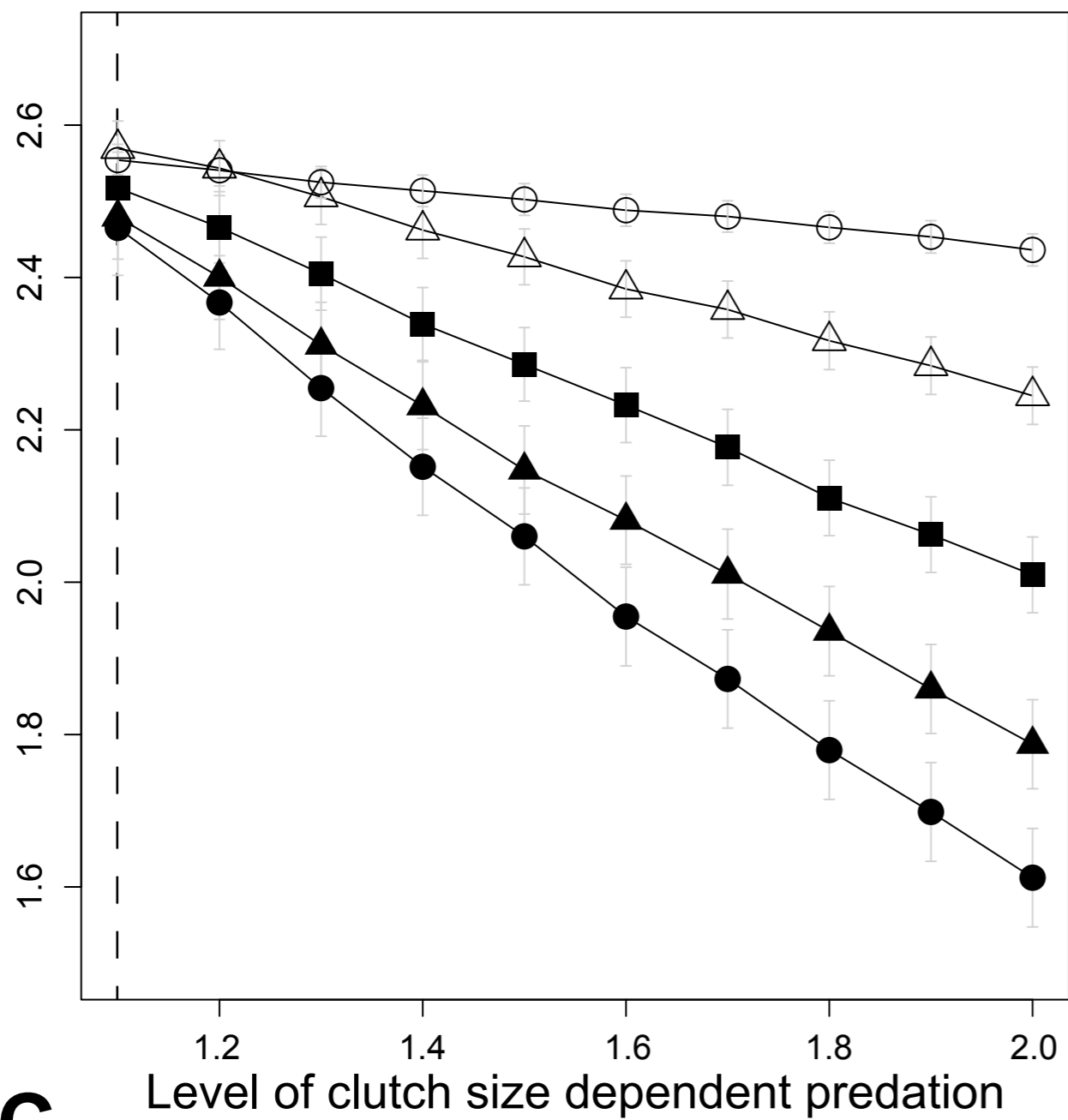

**B**

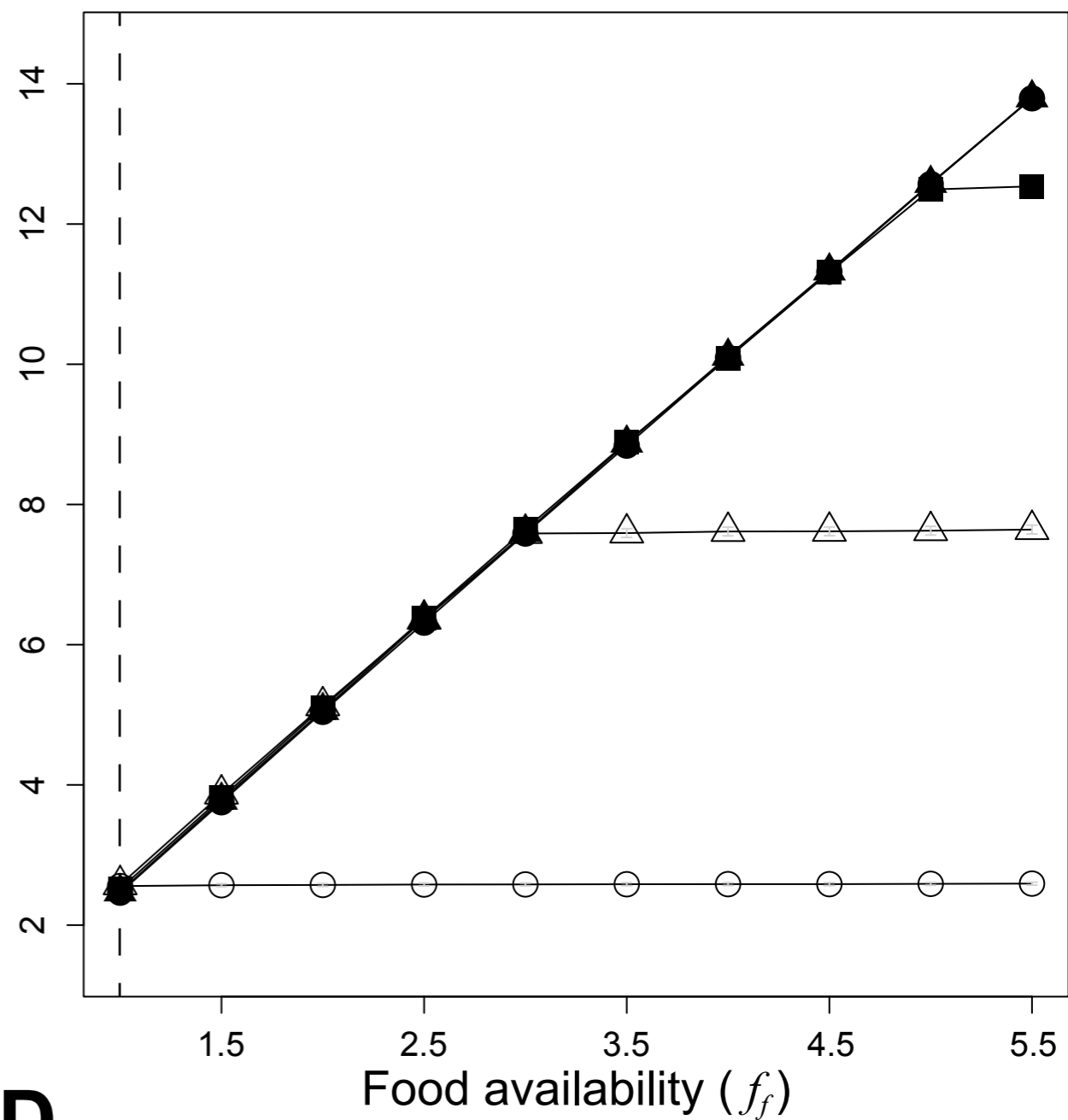

**C**

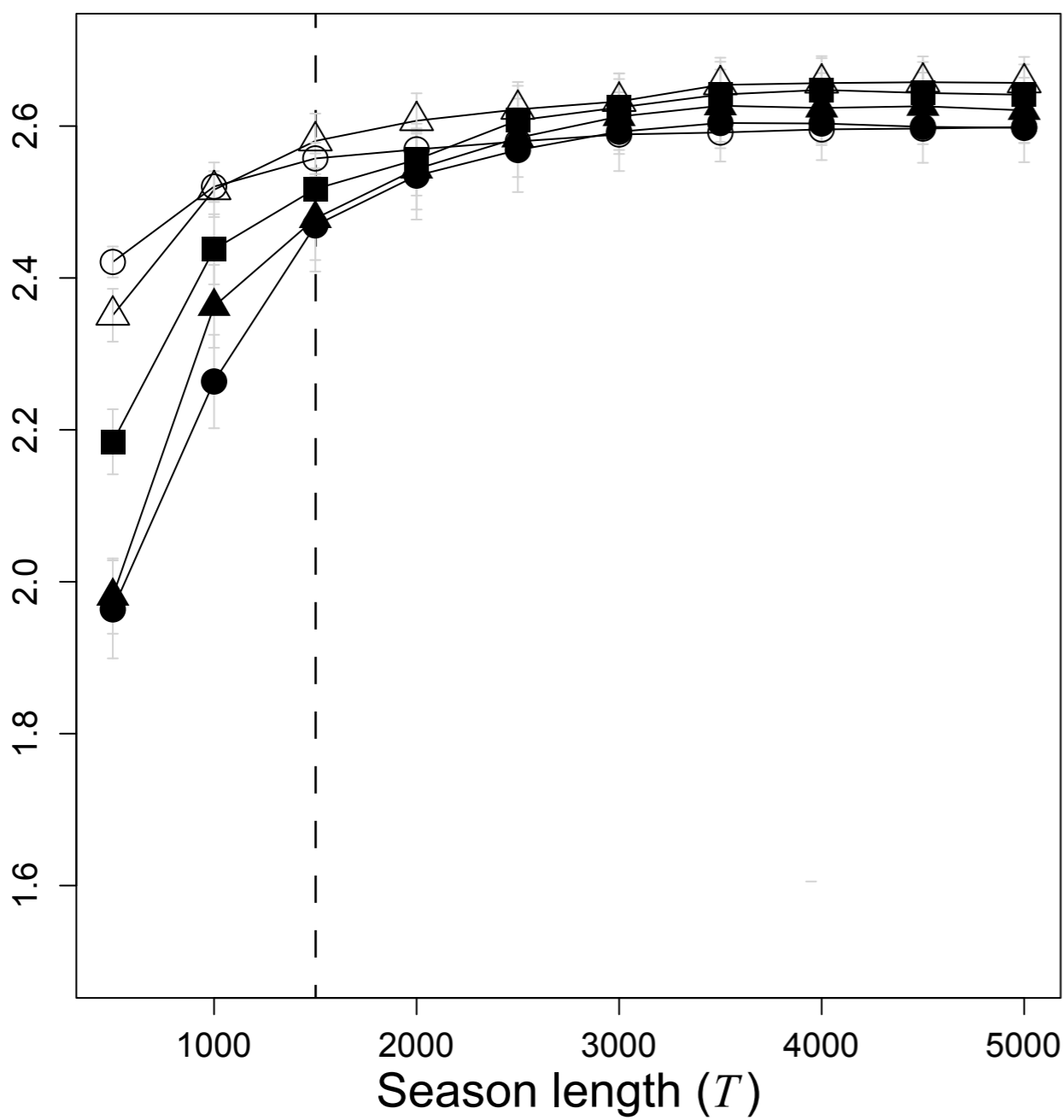

**D**

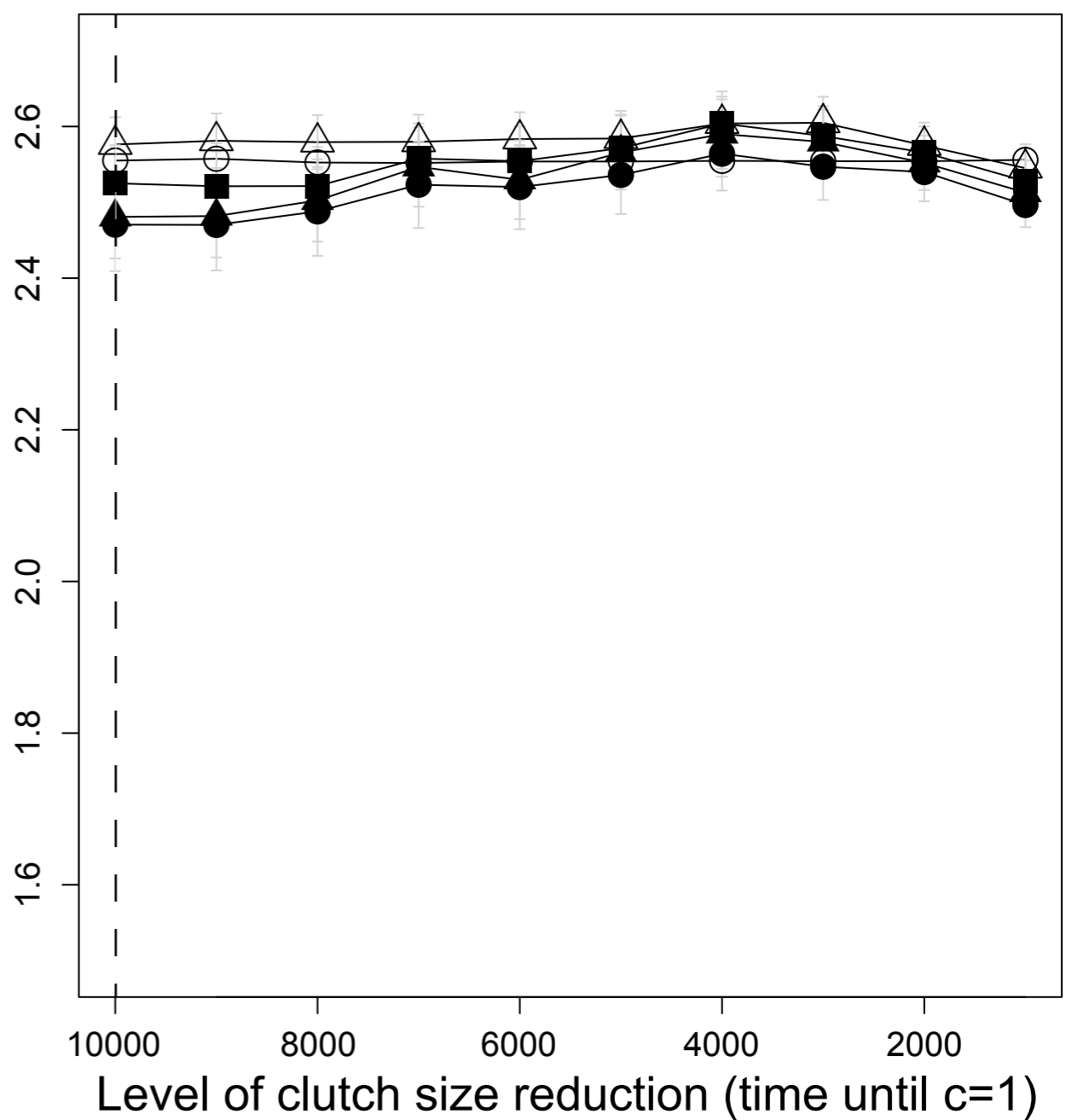

Clutch Size ○  $c=1$  △  $c=3$  ■  $c=5$  ▲  $c=7$  ●  $c=9$

Supplement: Supplementary file 2 [file ECE3-8-8803-s002.pdf]
