# Supplementary material for: Multitasking and the evolution of optimal clutch size in fluctuating environments
Source: Ecol Evol. 2018 Aug 7;8(17):8803–17. doi: 10.1002/ece3.4364 (PMC6157677; doi:10.1002/ece3.4364)
Supplement: Supplementary file 3 [file ECE3-8-8803-s003.docx]

**Supporting Information**

*Clutch size reduction through breeding season*

We assume a fixed clutch size strategy for simplicity in the main text. Here, we relax this assumption and assume that clutch size can decrease later in a breeding season. Specifically, the size of reduction is proportional to length of season elapsed, that $c_{r}= round(c-t/2000)$, where $c_{r}$ is the adjusted size, $c$ is the original size, and $t$ is the duration after breeding season began. If $t$ is greater than 2000, $c_{r}$ is unity. We find that the difference between fixed and reducing clutch size strategies is more pronounced under the larger clutch size strategy. Interestingly, opposite directions of change occur after incorporating size reduction in shorter (Fig. S1A, increasing) and longer breeding seasons (Fig. S1B, decreasing). Once again, this can be explained by the excessive reserve, as reduced clutch sizes produce less excessive reserve and make the larger clutch strategy have similar numbers of offspring to the smaller clutch size strategy. Moreover, reduced clutch sizes also lower the energetic efficiency of the larger clutch strategy and decrease its mean offspring number. Hence, if there is no additional factor, clutch size reduction is beneficial in short breeding seasons but ineffective in long seasons.

*Comparison of weighting in each factor to the selection of clutch size*

Finally, we summarize all of the factors mentioned in the text and explicitly compare the amplitude of their effects. Four factors are compared: (1) Optimal clutch size decreases (from 3 to 1) when the level of clutch size dependent predation increases (Fig. S2A). Clutch size dependent predation selects for the smallest clutch (clutch size of 1) because it always faces the lowest risk of nest failure. (2) When food availability rises, optimal clutch size increases (Fig. S2B) because when food is more abundant, the recovery time between reproductive attempts can be reduced. (3) As described in the text, optimal clutch size increases (from 1 to 3) when season length becomes longer (Fig. S2C). Yet, this impact is less significant when the breeding season length is longer than 3000 time steps due to the excessive reserve being less important in longer seasons. (4) The difference in offspring number becomes smaller when the level of clutch size reduction increases (Fig. S2D). However, the trend is not clear in the current setting where season length is not very short (i.e. 1500 steps). Compared to the previous section, we may say that clutch size reduction only has a greater impact in short seasons (Fig. S1A). Considering all of these factors together, we conclude that the latitudinal trend in clutch size variation can be generated by food availability (i.e. seasonality, with higher food production during breeding season) or clutch size dependent predation (i.e. lower level of size dependent predation in temperate region), and that the elevational trend in clutch size variation can be generated by breeding season length. Thus, the fecundity gradient paradox could be explained by multiple factors (i.e. food availability, clutch size dependent predation and breeding season length) along the different environmental gradients.

**Figure legends of supporting information**

Figure S1. Comparison of constant/decreasing clutch size through breeding season in shorter (A, 500 steps) and longer breeding season (B, 10000 steps). Lines with empty circles (i.e. without reduction) resemble the result of Fig. 4A.

Figure S2. The synthetic model incorporating all factors potentially explaining the evolution of clutch size. The optimal clutch size can be viewed as a function of four parameters: season length (*T*), food availability (*f_f_*), the level of clutch size dependent predation risk, and clutch size reduction. We alter the weighting of each parameter one at a time in each panel to demonstrate their effects on offspring number under each breeding strategy. Dashed lines indicate the simulations with the same parameter setting (i.e. season length is 1500 steps, food availability is 1, level of clutch size dependent predation is 1.1, and all strategies reduce clutch size to 1 at 10000 time steps). Specifically, the nest failure rate under clutch size dependent predation linearly increases with increasing clutch size, and the “level” indicates how much times is the failure rate of clutch size of 10 to clutch size of one. On the other hand, all clutch size strategies reduce clutch size by the same proportion throughout the breeding season, and the “level” of clutch size reduction describes how fast that all strategy reduce to clutch size of 1. As a matter of comparison, the level of size dependent predation in the text is 2.0 (Fig. 5), and the time until clutch size reduces to one is 2000, as noted the in previous section (Fig. S1).
